# Conceptions of Contraceptive Use in Rural KwaZulu-Natal, South Africa: Lessons for Programming

**DOI:** 10.3390/ijerph14040353

**Published:** 2017-03-28

**Authors:** Catherine Ndinda, Tidings Ndhlovu, Nene Ernest Khalema

**Affiliations:** 1 Human Sciences Research Council, Private Bag X41, Pretoria 0001, South Africa; 2 School of Social Sciences (Sociology), University of KwaZulu-Natal, KwaZulu-Natal Dalbridge 4041, South Africa; 3 Department of Economics and International Business, Faculty of Business and Law, Manchester Metropolitan University, Manchester M15 6BH, UK; t.ndhlovu@mmu.ac.uk; 4 Graduate School of Business Leadership, University of South Africa, Midrand Johannesburg 1685, South Africa; 5 School of Built Environment and Development Studies, University of KwaZulu-Natal, KwaZulu-Natal Dalbridge 4041, South Africa; khalema@ukzn.ac.za

**Keywords:** contraceptive use, sexual and reproductive health and rights, Kwazulu-Natal, focus group discussions, socio-cultural dynamics, political economy, gender

## Abstract

Community family planning programmes in South Africa arose from the controversial apartheid history of controlling the African population while encouraging the growth of European migrant population. Post-apartheid population policies shifted away from population control to aligning policies to the global agenda that placed emphasis on the link between population and development. The focus on population and development polices in post-apartheid South Africa is on social equality, justice and peace rather than controlling sections of the population. Given the shift, this paper interrogates the conceptions of contraceptive use among rural communities in KwaZulu-Natal. Our primary objective is to understand the dynamics surrounding access to and use of family planning services in peri-urban and rural areas of KwaZulu-Natal. Using focus group data, the findings of the study suggest that different social categories interact with the family planning programmes differently. How teenagers and married women perceive the value of family planning differs. Gender differences regarding the use of condoms are also evident. The paper attempts to grapple with the non-use of condoms despite the knowledge that these prevent pregnancy and provide protection from sexually-transmitted diseases. The contribution of this paper lies in its identification of socio-cultural factors and the political economy underlying the different attitudes towards contraceptive use in rural KwaZulu-Natal.

## 1. Introduction

The dominant neoliberal viewpoint on population growth has typically characterised family planning as a tool for controlling purportedly overpopulation in countries such as Bangladesh, Ethiopia and Mexico, while the state provides appropriate education and financial incentives such as tax breaks to reduce fertility [1,2,3,4]. Moreover, the state’s facilitation of individual choice (regarding desired fertility) and opportunities arguably ensures that developing countries eventually converge with developed countries at latter stages of development [5,6]. In the meantime, proponents of this approach add that celibacy is likely to threaten the preservation of humanity. For example, in Apartheid South Africa, the national family planning programme that was passed in 1974 sought to provide contraception in order to reduce the black population while actively encouraging the growth of the white population (Department of Social Development (DOSD) [7]. Paradoxically, the ideological standpoint that informed this approach linked population growth with reducing poverty of the masses, yet institutionalized racism and exclusion from opportunities went against freedom of choice and convergence [2,3,8]. Despite linking incentives to fertility rates, population controls led to constrained freedoms as the black population lost their ability to exercise control over various aspects of their personal lives, including control over social reproduction. While the infrastructure was inadequate, designed, as it were, to cater for the minority white population so that the vast majority of the black population was unable to access adequate water, the 1984 Population and Development Programme (PDP) still continued to connect reduction of fertility (via family planning) with water scarcity [9].

This approach has been contested elsewhere. Concepts such as freedom of choice have been challenged, and human ingenuity (as shown in Thomas Malthus’ time and subsequently) have demonstrated that population growth is not necessarily linked with economic growth and human survival. Thus, some approaches on population have shifted emphasis towards development issues. In Mexico, for example, the very low uptake of contraceptive services in rural areas compared to urban areas forced the government to introduce “a new programme of reproductive health in which it legitimized both concerns (i.e., population control and reproductive health). In this way (similarly to Ethiopia), population control is retained as a legitimate policy goal with respect to modernization of the country, whilst at the same time the concerns of reproductive health advocates are also addressed through the adoption of an informed choice agenda” [10]. Given questions of contraceptive cost effectiveness, the Bangladeshi government, like the Mexican government, also turned its attention to “individual and cultural beliefs/factors” and gender issues to improve health and social wellbeing [10,11].

In South Africa, the democratic government (1994) also shifted its conceptualisation of family planning from population control to redressing past neglect and violations of human rights. The Department of Social Development (DSD) became concerned with population and development issues, while the Department of Health (DOH) focused on general health matters including sexual reproductive health. Aligning family planning to the global development agenda was aimed at a progressive agenda for social equality, justice, development, peace, as well as the Millennium Development Goals (MDG) targets on gender equity, and sexual reproductive health [12,13].

This paper concerns itself with yet another, albeit less discussed, approach to population and family planning, that is, a critical synthesis of cultural, social, political and economic factors that mediate or interact in specific ways with the broader society, its institutions, and power structures [14,15,16]. We seek to understand how people in peri-urban and rural KwaZulu-Natal (KZN) conceptualise and perceive family planning and contraceptive use, as well as access to reproductive health services. The purpose of the analysis is to provide detailed information about these realities and deepen the analysis beyond “what is known” or said, that is, carefully distinguish how peri-urban and rural people particularly perceive barriers to access from their social and cultural standpoint. In this endeavour, it is also important to heed “C. Wright Mill’s fierce warning: ‘theory without data is empty, data without theory are blind’” [17].

The paper begins with a conceptual framework that lays emphasis on culture, identity, gender and power. The sociocultural context of KwaZulu-Natal shows how knowledge and practice about contraceptive use are allied to specific cultural and ideological viewpoints. The methodology and findings are followed by a discussion and conclusion that explores the policy implications in a broader international context.

## 2. Population and Development: A Conceptual Framework

Debates on political, social and economic implications of population growth and control have been raging ever since the Enlightenment. Hutcheson (1755) [18] urged individuals to exercise restraint with regards to child-bearing, and warned that celibacy could undermine replacement levels of fertility and threaten the very existence of the human race. He was concerned with the size of the population, especially the possibility of below-replacement levels of fertility (BRF), as well as the kind of people that could emerge if emphasis was not placed on educating and socializing children to arguably become “good citizens” [18,19,20]. For his part, Robert Malthus (1798) [21] predicted a doomsday scenario in which “human population, when unchecked by political and moral constraints, grows faster than food supply” [22]. To effect population control, Malthus felt that “population must *always* be kept down to the level of the means of subsistence”. While his prognostications proved unfounded given the technological advances in agriculture and manufacturing in the subsequent century and a half, the 1970s gave heart to neo-Malthusians, as population growth was accompanied by energy and environmental problems (ibid).

It is against this background that the neoliberal view is developed on the basis of rational individuals who make choices and, through consumption, maximise happiness subject to income and technology constraints [6,22,23]. Given their biological dispositions, and the responsiveness/sensitivity of fertility to financial incentives, they make decisions about family size preferences by weighing up the cost and benefits of desired children relative to contraceptives, similarly to buying commodities. Communal ties are correspondingly undermined and broken up; indeed, old regulatory mechanisms are dissolved and replaced by new moral codes that have a slant towards “greater tolerance for individual choice” [23]. Education (to counter misinformation about contraceptive methods), easier access to contraceptives, and the extent to which barriers such as cultural influences are eroded or reduced, arguably aid individuals’ rational decision-making process.

Thus, government intervention is confined to providing incentives that influence beliefs, traditions and behaviours concerning fertility desires; as well as opportunities that enhance an individual’s self-fulfilment [1,6,19,20,23,24,25,26,27].

The alternative social organisation perspective contends that the state plays a more active role in its *carrot* (e.g., funding and championing family planning programmes) and *stick* (putting pressure for changes in attitudes, habits via family laws, taxation) policies. While some theorists incorporate institutional and cultural factors into neoliberal views, others contend that ‘Westernization’ (the diffusion of ideas, including the introduction of the nuclear family) explains why fertility falls in societies that have hardly been touched by ‘modernization’ (structural economic organisation) [1,2,4,17,23,28,29]. With regards to family planning methods, people arguably take into account a whole host of factors besides financial incentives, namely; desired family size, abstinence and breastfeeding, postponement of first pregnancies and marriage, the extent of service delivery, including contraceptives, and peer/societal pressures. They value income or family resources as much as their wellbeing, and regard enhancement of their capabilities or skills as improving their chances of accessing health, including family planning, and education. They also take account of implications for human rights, cultural surroundings and freedom to determine the course of their lives, giving voice to their preferences through democratic and political deliberations [9,30,31,32,33].

The third, and often neglected politico-cultural-socio-economic perspective, takes a step further in providing an understanding of population growth, contraceptives and/or family planning within the context of “political and economic structures, and social and cultural forms, of the people amongst whom they are found and (perhaps) used, and the local realities that mediate and ultimately define a global phenomenon such as contraception. Cross-cutting all this is the role of ‘power’ in human relationships and organizational structures” [34]. Historical specificity, cultural and ideological justification of the role of women in the family, as well as the institutional context of family and labour policies that reflect complex class, gender and racial oppressive relations, all play a part in shaping the way people perceive family planning programmes [8]. Simply put, the extent of male and (extended) family involvement and the ideological context concerning reproductive health must be taken into account alongside the *gender-equity dividend* which has far-reaching implications for decisions on fertility [5,11,29,35].

## 3. Methodology

### 3.1. Research Design

Research design is a critical element in conducting social research and the current study employed the cross-sectional research design that entails collecting data among study participants at specific point in time [36]. While noting the limitations of the cross-sectional research design compared to others such as case-control or cohort studies, the cross-sectional design was useful in providing a snapshot of perceptions and attitudes related to contraceptive use, teenage pregnancies and sexual and reproductive health issues more broadly.

Given the multidisciplinary nature of this study and the need “to deal effectively with the complex interplay of culture, power and technology cross-culturally” [10], a mixed methods approach was considered to be appropriate in facilitating the findings of the study. Secondary analysis of quantitative data was conducted to establish the sexual and reproductive health status in rural KwaZulu-Natal. The qualitative arm of the study comprised of focus group discussions (FGDs) that were conducted in rural communities, as reported in the latter part of this paper.

### 3.2. Recruitment of Study Participants

Focus group participants were recruited using the snowball technique. In the communities where the study was conducted, the researchers made contact with community representatives and shared the recruitment criteria that had been developed prior to visiting the study sites. The eligibility criteria required FGD participants to be over 18 years of age, and each FGD had to have a mix of ages ranging from 18 to over 65 to ensure that there was no bias based on age. FGD participants comprised of young people (18–24 years), middle-aged participants (25–34) older participants (35–59) and pensioners (60 years and above). Prior to commencement of fieldwork, we initially sought an equal number of homogenous male and female FGDs, but the conditions in the field resulted in mixed FGDs and an unequal number of male and female FGDs. The FGDs lasted between one and half to two hours each.

The focus group discussion (FGD) guide was developed in English and translated into isiZulu. The instrument was also back-translated to ensure consistency in the meaning of the terms used. The Zulu translation was shared with stakeholders for comment and to ascertain that the meaning of the Zulu translation was consistent with the meaning in the English translation.

### 3.3. FGD Process

The FGD included questions about knowledge and awareness of sexual and reproductive health determinants (SRH), access and barriers to SRH, magnitude of HIV and AIDS, teenage pregnancy and demographic information of the study area. Each focus group was moderated by a facilitator and the note-taker who took notes of the discussion and ensured that the discussion was electronically recorded. Each of the recordings was downloaded and transcribed verbatim in isiZulu. A translation into English was done below each Zulu sentence. The researchers validated the translations and had discussions with some of the participants regarding the accuracy of the translations to ensure that the meaning of the FGDs in isiZulu were retained in the English translations.

A total of nine (9) focus group discussion groups (FGDs) were conducted in isiZulu. Five of the FGDs were mixed FGDs, one a homogenous female FGD, and three all-male. The FGDs were stratified in terms of region (rural, urban, peri-urban) and by gender. The researchers limited the age of participants to 18 years and above since 18 is the age of consent in South Africa. The study was reviewed by the Ethics Review Board in line with the principles of ethical research to ensure the protection of participants against any harm and was granted approval (Ethics Committee Protocol No REC7/16/10/13).

### 3.4. Data Management and Analysis

The textual data was subjected to content analysis and constant comparative methods, with the NVivo Software drawing out the key themes emerging from the study. The constant comparative method entails making comparisons across data in order to explicate the relation between the different elements of the raw information. Analysts such as Hsieh and Shannon [37] define content analysis as ‘a research method for subjective interpretation of the content of text data through the systematic classification of coding and identifying themes or patterns’ (p. 1278). While contraceptive use was not one of the key questions posed, it nevertheless emerged as a theme under teenage pregnancy.

### 3.5. Characteristics of Study Participants

The demographic characteristics of the study participants were reflective of the provincial demographic profile. About 81% of the population in KZN speaks isiZulu (Statistics South Africa, 2006) [38] and identifies with the Zulu-nation. Among Africans who do not identify as Zulu within the province, knowledge of conversational isiZulu is common particularly if they belong to the Nguni language groups (Xhosa, Swati). Data on education suggests that about 37.7% of the population over 20 years has 12 years of schooling compared to 40.6% of the population within the same age category in Gauteng which had 12 years of schooling [38].

Like the study participants, a large proportion of the population in KwaZulu-Natal identify as Christians and a majority affiliate with the Zion Christian Church (ZCC). By 2004, about 1,179,610 members of the KZN population reported affiliation to ZCC and another 925,490 reported affiliating with the Roman Catholic Church [38]. A large proportion of Africans in particular affiliate with the Independent Christian Churches such as Shembe and a small proportion (20,959) affiliate with the African Traditional religions (Statistics South Africa 2004). A large proportion in KwaZulu-Natal identify as not belonging to any religion (1,772,534 individuals) [38]. It is worth noting that religious affiliation does not necessarily correspond to active participation in religious rites on a weekly basis but, rather, represents an element of belief and faith in the ultimate reality greater than self. Yet, as Mbiti [39] argues, Africans are notoriously religious and spirituality is part and parcel of their identity. The religiosity of the study participants like that of the general KZN population was reflected in the language used and the ways in which ideas about contraceptive use and family planning in general were expressed.

### 3.6. Study Areas

Despite population growth rates in KwaZulu-Natal being stagnant at 0.7% between 2001 and 2011, total population stands at around 10.5 million [40,41], the second most populous province in South Africa after Gauteng. KwaZulu-Natal occupies 92,100 km^2^. There are eleven districts in the province, namely: *Ugu*, *UThukela*, *UMgungundlovu*, *UMzinyathi*, *Amajuba*, *Zululand*, *UMkhanyakude*, *UThungulu*, *ILembe*, *Sisonke* and *eThekwini*. The most densely populated municipality is *eThekwini*. Between 1996 and 2001, the population growth rate stood at 2.2%, subsequently falling to 0.7% between 2001 and 2011 [40]. Fertility rates also give us a clearer understanding of the overall conception of family planning in KwaZulu-Natal. Although fertility rates fell from 3.03 children per woman in 2001 to 2.60 in 2009, there were still higher than the national average of 2.38 children in 2009 [42].

In terms of the sexual and reproductive health, there have been perceptible changes in KZN. In the 1996 census, the average household size was 5.1 individuals per household, with 3.1 children per household, subsequently falling to an average of 2 children in 2011 [40]. More than two-thirds of the population have never been married, while about a quarter are reported to be currently married or cohabiting, and less than 5% of the population is reported as widowed (1%) and separated or divorced (2.9%) [40].

Notwithstanding this, the total fertility rate in KwaZulu-Natal (KZN) is relatively high among the African population. Notable is the fact that for every 100 males, there are 108 females and about two-thirds of the population is aged between 15 and 64, whilst the population aged below 15 years of age accounts for about a third of the total population [40]. Those who are 65 years and above account for about 5% of the population [40]. The high incidence of teenage fertility has not resulted in increased contraceptive uptake. If anything, serial monogamy or multiple concurrent sexual relationships are the norm. In the meantime, the province continues to grapple with the dilemma of disseminating appropriate/sensitive messages about safe termination of pregnancies, whilst seeking ways of addressing infertility and reproductive cancers for both males and females.

While entitlement to reproductive health services is certain, access is correspondingly limited, particularly in remote areas, as evidenced by the low Couple Years of Protection rate and data on condom use among males. CYP, or contraceptive prevalence rate, estimates protection provided by family planning (FP) services during a one-year period, based upon the volume of all contraceptives sold or distributed free of charge to clients during that period. CYP measures the volume of programme activity from data routinely collected through programmes or projects, or service delivery mechanisms (clinics, community-based distributors, social/commercial marketing). The CYP rate for KZN is 37.5, compared to the national average of 37.8. According to the Department of Health, the national male condom distribution rate stands at over 350 million annually [42]. The male condom distribution rate measures the number of condoms that are distributed yearly to males of 15 years and older by the Department of Health via public health facilities and other outlets in the districts. Over the period 2008–2009, an average of 12.3 condoms per male were distributed in South Africa to males aged 15 years and above [42]. The Department has since increased this rate. Whilst a robust distribution campaign has created the impression of easier access to free male condoms, usage among males is moderate. Subjected to cultural and societal pressures, men between the ages of 15 and 49 [42] continue to make crucial decisions about reproductive health practices such as condom use.

## 4. Results

The profile of participants who contributed to the focus groups in this paper embody the characteristics of the provincial demographic profile. Where there might be differences, this is largely explained by how the Zulu culture is practiced in the different districts and localities as indicated in the discussions. In other words, participants were all African and were drawn from different districts in KZN and localities in each districts, hence the diversity of views presented in this paper. They were drawn from both peri-urban and rural areas. The specific areas where the study was conducted are shown in Table 1. Although the places might be in different geographical areas within the province, the provincial demographic and cultural profile is reflective of these local areas.

Complexities concerning access to relevant family planning information, distribution of contraceptives and their usage, were all raised in focus group discussions in the province of KwaZulu-Natal (KZN). Participants were more knowledgeable about modern family planning methods than traditional ones. They reflected on the applicability and resurgence of indigenous methods of family planning such as virginity testing (*ukuhlolwa*), rites of passage and so forth. A debate on how these could be integrated with modern methods dominated the discussions. Abstinence and male condom use were commonly cited as preferred family planning methods, followed by pills and, to a lesser extent, female condoms. In KZN, where there is the lowest contraceptive usage, participants mentioned access to most contraceptives as a challenge. This may be due to cultural and religious factors, and perhaps because a relatively small number of women use modern methods of birth control. Cultural, gender and power relations arguably have implications for reproductive health rights of women in KZN. To understand the high teenage pregnancy rates and inconsistent or low contraceptive uptake, we conducted an in-depth study in peri-urban and rural KZN in 2013.

Each gender group proceeded to blame the other for lack of contraceptive knowledge. Nevertheless, most groups agreed that family planning services were available, although constraints to accessing these services were individual, cultural and/or engrained within the health system. Given the unfavourable experiences of China, India, Mexico and Ethiopia, the government had gone so far as to make different types of contraceptives available, but was nevertheless wary of forcing eligible people to use them.

**Participant #21:** Yes there is a way and it is available at the clinic. If you go to the clinics there are sisters who stand in front of us and explain [that] there are pills, injections and currently [that] there is a new way. That procedure called the loop was used in the olden days by our grandparents (FGD_Mixed_Ulundi).

**Participant #21:** Yes it is a prevention method. It’s something that the woman must always have on at all times (FGD_Mixed_Ulundi).

### 4.1. Knowledge about the Use of Family Planning in the Community

In both urban and rural areas, participants reported that there were programmes that taught people about birth control, and that couples could choose their own form of contraception. The discussants were emphatic that partners should agree on the number and spacing of children, but felt that peer pressure could not be ruled out when it came to reluctance to use contraceptives and the resulting uncontrolled births. It was stated that adolescents dated and experimented with sex at an early age. Despite the availability of family planning services, teenage pregnancies remained a challenge due to the low uptake by the youth. Availability of and high levels of awareness of contraceptives among the youth did not translate into changes in sexual behaviour or health seeking behaviour. Participants expressed frustration that awareness about contraceptives was not matched by prevention of unwanted pregnancies:

**Participant #5:** There have been campaigns … that mentioned that people should go to their nearest NGOs so that they can be educated. The youth are very smart, before you do something you have to know what’s going to happen after that and there are contraceptives and things like that (FGD_Mixed_Stanger).

While discussants emphasised the role played by parents in birth control and prevention of teenage pregnancies, views differed on how to ensure change in youth behaviour to prevent teenage pregnancy through the uptake of contraceptives. Some study participants reported that parents did talk to children about sex and sexuality. In peri-urban KZN, fathers encouraged boys to use condoms. In rural KZN, on the other hand, parent–child communication was cited as a challenge in promoting responsible sexual behaviour. Instead, the perception was that parents communicated about sex and sexuality in a reprimanding manner. As a result, it was contended that the message regarding responsible sexual behaviour and preventing unwanted pregnancies was often lost to the youth. A participant argued that there were no known methods of educating the youth about sex and reproductive health. Despite most participants agreeing that sex and reproductive health education was important, participants were emphatic that parents traditionally do not discuss sex with children, as this is considered disrespectful and outrageous. When children initiate such discussions, corporal punishment often follows since sex is a taboo subject in Zulu culture. Rural participants argued that discussions about birth control between parents and children occurred after a girl had given birth.

**Participant #17**: …We as parents grew up in a different time as our children, a time where we did everything “recklessly”. The time our children are growing up in is right, everything is easy for them. They must be alert to the things they are told by their parents at home and they also watch Generations and every other soapie, so they should know that 1 + 1 = 2 and 1 and 1 is 11. Thank you. (FGD_Mixed_Stanger).

**Participant #29:** ...I hear Mama XX saying we need to test our children, we do that, the government must visit schools. When a child starts menstruating, take them for contraceptives because it’s hard for us as parents to encourage our children to use contraceptives because it’s like you saying go ahead and have sex, just use contraceptives. So it’s better if the government visits schools and assists the children there. When a child is 15 years, they must start contraceptives. Our children have failed us, we cross-examined them in pairs—we do not know what to do as parents (FGD_Mixed_Ulundi).

Although it was acknowledged that adolescents were having their first sexual encounters at an early age, parents were at a loss on how to address the problem. Cultural norms that constrain parents from discussing sex with their children persist. While some parents are of the view that adolescents should use contraceptives to avoid pregnancy, there is ambivalence about following this through for fear of it being interpreted as condoning sexual activity among teenagers. Since discussions on sex are taboo, parents are reluctant to broach the subject with children [43]. In the circumstances, parents delegate responsibility for sex education and contraceptive use to the State.

### 4.2. Condom Use

Participants in a male FGD in peri-urban KwaZulu-Natal argued that although condoms prevent pregnancy and protect against sexually transmitted infections, girls did not like using them. It was contended that: sex with condoms was not pleasurable; sexual partners (women) complained that condoms were painful; and sexual partners (women) did not like using condoms because they allegedly wanted to access child grants.

However, one of the male participants also noted that conflicting and contradictory messages from those in authority often left young men confused about condom use. Some participants insisted that condoms and abstinence were not effective measures against unwanted pregnancy because condoms were sometimes torn during sex. Yet others strongly articulated the public health message that condoms protect against sexually-transmitted infections and prevent unwanted pregnancies.

**Participant #21:** although condoms are not 100%, I think we should carry on preaching that verse (FGD_Mixed_Stanger).

These apparently contradictory views suggest that opinions are also shaped by whether or not people had experience with condom use. In some quarters, consistent government messages about the importance of condom usage to prevent HIV and AIDS did not seem to be getting through. For example, participants in rural KZN argued that condoms were responsible for the increase in pre-marital sex, their availability in beer-drinking venues (taverns) cited as a case in point. While making condoms more widely available can protect people from unwanted pregnancies and against sexually-transmitted diseases, the moral and cultural argument, including the belief that population control is the primary objective, must not be summarily dismissed in a bid to push for and promote condom use by family planning campaigners. It was a genuine concern in rural KZN.

For their part, the study participants were convinced that contraceptives loosen the vagina and leave women with stretch marks, hence the reluctance to use them. These findings are consistent with studies conducted in the same region, including the belief that contraceptives reduce sexual pleasure [44].

Although the government made contraceptives available in local clinics in rural KwaZulu-Natal, it was incumbent upon partners and individuals to choose their birth control measures. Societal beliefs play a part in influencing views concerning contraceptive use.

### 4.3. Culture and Birth Control

As indicated earlier, participants in rural KZN observed that birth control in their community was mostly used by youngsters who had already given birth and were anxious to prevent further pregnancies. They also noted, with a heavy heart, that condom use in particular was necessary to prevent young girls from getting pregnant while still at school. Community health workers (referred to as nurses) provided information on birth control during their home visits. Although participants claimed to be aware of injectable contraceptives, most of the discussions were clearly influenced by family planning campaigns which emphasise condom use as a form of contraceptive. Such an approach seeks to tackle the high levels of HIV and AIDS in South Africa and KwaZulu-Natal in particular, and also promote protection against unwanted pregnancies. However, one long-held view is that having one child puts parents at a disadvantage. If the child dies, the woman is effectively stripped of her status within Zulu culture.

Moreover, while the Department of Health (DOH), in partnership with faith-based organisations, disseminates information about condom use in a bid to reduce new HIV and AIDS infections, community members seem to associate condoms with ‘white people’. This suggests that messages may be contradictory and not as sensitive to local contexts as they should be. As the extracts suggest, religion of one sort or another is an important social institution among the rural population in KwaZulu-Natal. Communicating messages about contraceptive use in the Church removes the binary between what the State advocates in terms of family planning and the message of the Church that advocate abstinence before marriage. State programmes advocating condom use may thus be accompanied by silence or ambivalence from the church regarding that particular form of contraception, thus, creating confusion in people’s minds. Cultural factors also play a major role in how far people are receptive to family planning messages.

**Participant #31:** what hurts us the most is that we think that the condom is only for white people as they discovered it. In churches, condoms are not spoken about, so churches should start educating people about condoms. There should be workshops in church that educate people and [nurses that] work for the department of health should educate the people (FGD_Stanger).

While participants were reluctant to acknowledge the influence of culture in their fertility decisions, their responses suggested that culture played an important role in explaining why family planning did not work. Study participants argued that women had to bear children to prove their worth. Not only is giving birth important, but bearing sons increases the value and worth of a woman in Zulu culture. When the first child is a girl, a woman is unlikely to go on contraceptives as societal pressure dictates that she tries again until she bears a son. This means that women can end up with more children than they planned for. Where women did not bear sons, their husbands were likely to marry second wives in an attempt to have sons who are considered to be heirs and bearers of the clan name.

**Particiant #23:** Maybe there aren’t any beliefs that are related to culture to us as people, that if you are to be married [you have to] have six kids; but if you have one kid, they would want to see if you are fertile or not (FGD_Ulundi).

**Participant #30:** You could say that our culture has an effect on [us] not using these things because black people tend to take religious matters too far. Like I would say that if the *verse* [referring to the verse is terminology that is used by church-goers] were to [be] spread just like the programmes that are shown on TV that talk about religion…TV has an effect especially on children. You see, when they see something on TV, they want to imitate it and not think about the consequences. Because most of the time they do not teach, so you could say that TV should play programmes that preach about the *verse* and how to care for yourself (FGD_Stanger).

Furthermore, the rural FGDs raised virginity testing without any prompting or probing. Virginity testing (or *ukuhlowa*) was identified as one of the most effective ways of preventing unwanted pregnancies among teenagers. Participants suggested that virginity testing enabled parents to advise girls on how to maintain their virginity after they had been tested. The practice of *ukusoma* (thigh sex) was also cited as one of the ways for preventing pregnancy in Zulu Culture.

**Participant #20:** there is something that was used by women that was called *ukusoma* (thigh sex); you find that she would sleep with a man but not actually be sleeping with him. She could press her thighs tight and the man would have intercourse with the thighs and then he feels it burning [in Zulu culture the use of polite language shows respect. In the extract, the expression that ‘he feels it burning’ is a polite way of describing arousal] then he will ejaculate [description of how thigh sex is practised]. It’s an easy way to prevent [pregnancies], it’s called *ukusoma*. Maybe things like that could come back, because the youth know nothing about it, or maybe only 10% know (FGD_Mixed_Stanger).

**Participant #29:** As there are so many diseases, when I was still a girl… when I started dating, there was a head girl who told me not to sleep with my boyfriend because I was going to get pregnant. I had to do *ukusoma*. But now our children do not even know what that is. That [is] what spreads the disease and everyone has the disease [the expression ‘the disease’ is a polite way of referring to HIV and AIDS] (FGD_Mixed_Ulundi).

*Ukusoma* or thigh sex is a method that was used traditionally to prevent pregnancy in Zulu culture. While older female participants confirmed having used *ukusoma* to prevent pregnancy in their youth, the practice seems to be on the decline. The lack of knowledge about *ukusoma* had not only resulted in teenage pregnancies but also in the spread of HIV, “the disease”. Culturally *ukusoma* has been successful in preventing unwanted pregnancies but whether the practice can help prevent HIV infections is not borne out by evidence and presents an avenue for further research.

The failure by health personnel to maintain confidentiality in providing family planning services was cited as a challenge. Participants noted that when the youth sought contraceptives, some nurses went to the extent of reporting teenagers to their parents for enquiring about contraceptives. Such attitudes among the health personnel are attributable to the setting in rural KZN. Traditionally, Zulu clans occupy specific areas within the province and, as a result, most members within villages and surrounding peri-urban areas are in one way or the other related through lineage or affiliation. Within such a setup, local health personnel know their clients, their religious affiliation and family and, given these circumstances, might feel obliged to report one of their “own” when they deviate from the norm that in the Zulu cultural context, requires that young women remain virgins until marriage.

As already indicated, bearing a daughter is no substitute since the only proof of fertility is bearing sons. Children, particularly sons, in Zulu culture confer status not only to men, but also women regardless of marital status.

Despite the government’s intention to encourage family planning, cultural beliefs and practices dictate that priority be accorded to bearing male children who are valued more than girls. Under pressure to bear sons, women end up with more children than they initially planned for.

**Participant #24:** It happens because I have seven children and I wanted a boy because it is a must to have one, so they can inherit and *forward the clan* [‘forward the clan’ is an expression that denotes continuing the lineage]. So it still happens (FGD_Mixed_Ulundi).

**Participant #25:** I am number 4 [meaning this is my forth child]. I have squashed it. It has been tough and I have been operated [on] for three children just because of this boy (FGD_Mixed_Ulundi).

**Participant #26:** Me too. I support this lady. I have six girls and the boy was the seventh child. I then tried again for another one then I got a girl (FGD_Mixed_Ulundi).

Zulu society is patrilineal and participants argued that, in line with culture, males maintain the family lineage and inherit from their parents. Traditionally, inheritance is bequeathed to male children as female children are expected to get married and contribute to the lineage of their husbands. Culturally, women neither inherit from their fathers nor their husbands, thus leaving them in a precarious state. It was emphasised over and over again that women were only validated once they bore sons. In their attempts to bear sons, women ended up with up to seven children, of which only one might be a son.

Given the cultural constrictions, participants in rural areas were more than willing to abrogate responsibility of sex and reproductive education for their children and delegate it to the state, amongst other organisations. They argued that birth control should be taught in schools, family planning centres, churches and in public places so that there were no unwanted pregnancies. They urged that more information be accessible to the general population. Since the importance of male children in Zulu culture is unquestioned, and marriage rates are low in South Africa, it is surprising that participants should suggest interventions similar to the “one child” policies that have now been abandoned by the Chinese government. Apparently, too many rights without responsibility were the main reasons for teenage pregnancies.

## 5. Discussion

The findings of our study suggest that the availability of condoms and corresponding knowledge that they prevent pregnancy and protect from sexually-transmitted infections does not always coincide with application of such knowledge. This is consistent with findings from Uganda where, despite high levels of awareness of and favourable attitudes towards contraceptives, their use remained low [45]. Our study found that men often regarded condoms as reducing sexual pleasure. This is in line with previous studies conducted in rural KwaZulu-Natal [44,46] that reported reluctance to use condoms despite the dangers of contracting HIV, and men equating their use to “eating a wrapped sweet”. Among women of child-bearing age, the reluctance to use modern contraceptive methods is driven by cultural factors and a sense of powerlessness. The high levels of unemployment and underemployment [47] imply that women have to depend on their partners for a living. Such dependence places women in a vulnerable position in negotiating condom use. As previous studies have shown [44], power relations are complex and power and powerlessness of women in sexual relationships depend on factors such as how they define the relationship with their sexual partners. Ndinda et al. [44] argue that women who are not in marital relationships are more able to dictate the terms of interaction with their sexual partners than those in marriage. However, this study adds that only those women who are economically independent can dictate the terms of their sexual relations with partners regardless of marital status.

Our findings showed that men were also convinced that young women were reluctant to use condoms because they wanted to access child support grants (CSGs) that were allocated to poor children by the Department of Social Development as a social protection measure. While such claims are familiar, they are not supported by evidence [48]. Teenage pregnancy has always been high, even before child grants were extended to the African population in post-apartheid South Africa. Teenage pregnancy rates were high in the 1980s but had declined by 1998 when the CSG was introduced [48]. Makiwane et al. [48] demonstrated that most recipients of the support grant were not young women as is commonly presumed but, rather, older women over the age of 31 years. The same study reported that only 20% of teenagers who bore children received the child support grant. What is important is that consistent messages about the importance of condoms for the prevention of sexually-transmitted infections and unwanted pregnancies be more sensitive to local situations and/or take account of cultural, gender and power relations. Indeed, explanations about the unwillingness of participants to use contraceptives, particularly condoms, can be linked to these factors.

The gendered attitudes towards contraceptive use and the tendency for one gender to blame the other for the failure to use them explain the failure to implement family planning programmes. In the case of Nigeria [49], a gender analysis showed that the critical variables in predicting the use of family planning among men were religion and ethnicity. Other predictors included exposure to the media, place of residence, and approval by spouse and partner communication. For women, the main predictors of contraceptive use were age, approval of female education and contraception, ethnicity, exposure to the media, communication with spouses and social support. Thus, the reluctance to use contraceptives seemed to be due to the dissonance between knowledge and practice. Arguably, this gap could be closed via the use of contextually relevant messages concerning contraceptives and their value in reproductive health. The message in family planning campaigns should include modules on parent-child communication to ensure that parents communicate important information on sexual behaviour in ways that are acceptable to and appropriate for the youth.

The South African approach to family planning is one in which the state provides sexual reproductive health services to those who seek them, so that they can plan and space births. Although contraceptives, notably free condoms, are available in clinics and specific public spaces, there is reluctance to use them, hence the unplanned and unwanted pregnancies particularly among teenagers. Moral and cultural arguments suggest that even when condoms are free, they are often placed where no self-respecting, church-going individuals would go or be associated with, such as bars (*sheebeens*, a term for informal bars in townships). South Africans are highly religious, with about 80% identifying as Christian and the rest being African traditionalists, Muslims and other faiths [50]. However, these views that were presented by some participants tended to disregard the fact that condoms were readily available in public clinics, toilets and elsewhere. As the findings in the current study suggest, the availability of services and contraceptives does not always translate into usage. Unlike the study in Nigeria [51] and Eastern Kenya [52], that were in line with neo-liberal views, decisions about failure to use contraceptives were not primarily based on ignorance, fear and/or unfounded cultural beliefs. Similar to studies in Western Kenya [53] Ethiopia [35], Uganda [45] and Greater Maputo (Mozambique) [54], cultural norms and beliefs matter as much as knowledge of both modern and traditional forms of contraception, media influence and perceived side-effects on the users [27,49]. Religion, life experience, urbanisation, (economic) status in society and gender norms also matter. Appropriately, providing family planning services requires a keen understanding of people’s beliefs, social context, practices and health-seeking behaviour. This is important in designing family planning messages rooted in the cultural context to ensure acceptability of family planning technologies and dispel the suspicion that population control is behind these programmes, while also helping us to understand why such programmes are often rejected. This paper recommends continuous training of health personnel involved in providing family planning services to ensure that details of youth who seek contraceptives are kept confidential. Moreover, health workers can be assessed for their sensitivity to and awareness of questions of confidentiality in the provision of family planning services.

Among married women, the reluctance to use modern contraceptives is associated with pressures to bear sons as this accords them honour in the family and community at large. Son preference generally refers to the “attitude that sons are more important and valuable than daughters” [55]. In the patrilineal Zulu culture, as in other African cultures, sons are more valued than daughters because they are the bearers of the family name and lineage. The status and honour of women is not only dependent on their child-bearing ability, but also becomes higher when they bear sons. Zulu culture is also polygamous (with the proviso that men be able to support their wives and children). For Zulu men, taking a second wife increases the possibility of having sons that can continue the family lineage. In the search for sons, the notion of family planning becomes secondary to Zulu women who are under family and societal pressure to prove their relevance by bearing sons.

The persistence of traditional beliefs that place greater value on sons is not unique to Zulus, but is also a cultural phenomenon across African communities [52,56]. In Western Kenya, gender preferences ultimately determine the number of children that couples bear [52]. Besides, son preference is not confined to African communities. It is prevalent in low-income countries and emerging economies in general [56,57,58]. Son preference results in what Clark refers to as “differential stopping behaviour (DSB)” [56]; in other words, couples stop bearing children once the desired gender composition is attained. Thus, “couples who have already attained the desired sex composition of children are more likely to stop having children than couples who have not reached their preference” [56]. In the case of rural Zulu women, DSB implies that child-bearing continues until they get sons and only stops when the desired number of sons has been achieved. Because of son preference in, for example, India [56] and China, population control policies resulted in selective abortion. Messages that are promoted by family planning campaigns should therefore include the importance of valuing children regardless of their gender and encourage the use of both traditional and/or modern contraceptive measures to ensure that the number of children parents have is based on whether they can afford to support them.

Under apartheid, the overwhelming message of the state was to control the African population while encouraging the growth of the white population. In post-apartheid South Africa, there is no evidence that son preference among Zulus and Africans in general has led to selective abortions. The state makes contraceptives available in clinics and elsewhere, but has avoided China’s forcible/selective “one-child” model. The Strategic Plan for Maternal, Newborn, Child and Women’s Health (MNCWH) (2012) indicates that state intervention in family planning has had limited success in providing reproductive health services, even with campaigns to encourage couples to use the dual prevention that entails the use of condoms and other contraceptives [59]. Given concerns about the high proportion of sexually active adults (15–49 years) living with HIV and AIDS, a rate that has remained steady at 17.3% since 2005 [60], adoption and promotion of this dual approach is designed to make in-roads into cases of sexually transmitted diseases. According to the MNCWH (2012), and supported by *The National Contraception and Fertility Planning Policy* and Service Delivery Guidelines [61], primary healthcare centres are required to provide the full range of sexual and reproductive health services and contraceptive methods.

Awareness of and access to modern contraceptive methods exist alongside the use of indigenous sexual and reproductive health practices and contraceptive methods in peri-urban and rural KZN. The methods cited in our study are virginity testing (*ukuhlolwa*) and *ukusoma* (thigh sex) [44]. Feminist attacks on virginity testing have not led to an end to these practices; indeed, our study finds that these practices are culturally acceptable methods of ascertaining reproductive health among teenage girls in KZN. *Ukusoma* allows partners to have non-penetrative sex, thus avoiding unwanted pregnancy [44,62]. In the circumstances, the survival of indigenous sexual and reproductive health methods of contraception have some utility in these communities. This finding is not unique to rural KZN. In urban Mozambique, women were reported to use a range of indigenous methods of contraception, ranging from herbs and amulets to charms believed to prevent pregnancy [54]. This paper argues that the implementation of reproductive health programmes needs to take into account the cultural context and practices of the target population if there are to be successful. Addressing indigenous methods of contraception and their merits would allow the target population to make informed decisions on contraception and fertility. As discussed at length in this paper, it is significant that interventions be sensitive to local situations and that the message takes account of social, political, cultural and ideological contexts.

## 6. Conclusions

In short, this paper set out to address the conceptions of family planning among participants in peri-urban and rural KwaZulu-Natal. We discussed theoretical underpinnings of family planning programmes internationally and locally and noted that literature points to spatial differences in the utilisation of family planning services within countries. The findings based on research conducted in peri-urban and rural KwaZulu-Natal are consistent with international literature that posits the persistence of cultural beliefs in influencing contraceptive uptake and family planning practices. The South African government takes a “soft-touch” approach towards implementation of family planning programmes. Our study argues that there is a need to take cognizance of cultural beliefs and practices in different contexts and pay more attention to women’s reproductive rights and/or power relations if programmes are to meet their stated objectives, or explanations are furnished for their failure.

## Figures and Tables

**Table 1:** Number of focus group discussions participants per district area.

District	Are of Study	Male	Female	Total
UThukela	Ladysmith	3	14	17
UThukela	Bergville	1	20	21
UMkhanyakude	Jozini	0	12	12
UMkhanyakude	Hlabisa	7	3	10
Zululand	Phongola	4	18	22
ILembe	Kwadukuza	8	0	8
Ilembe	iNdwedwe	8	0	8
Ugu	Port Shepstone	9	14	23
Zululand	Ulundi	6	10	16
		46	91	137
